# Formulation, Characterization, and Cytotoxicity Evaluation of Lactoferrin Functionalized Lipid Nanoparticles for Riluzole Delivery to the Brain

**DOI:** 10.3390/pharmaceutics14010185

**Published:** 2022-01-13

**Authors:** Maria Inês Teixeira, Carla Martins Lopes, Hugo Gonçalves, José Catita, Ana Margarida Silva, Francisca Rodrigues, Maria Helena Amaral, Paulo C. Costa

**Affiliations:** 1 Associate Laboratory i4HB—Institute for Health and Bioeconomy, Faculty of Pharmacy, University of Porto, Rua de Jorge Viterbo Ferreira, 228, 4050-313 Porto, Portugal; hamaral@ff.up.pt (M.H.A.); pccosta@ff.up.pt (P.C.C.); 2 UCIBIO—Applied Molecular Biosciences Unit, MedTech—Laboratory of Pharmaceutical Technology, Department of Drug Sciences, Faculty of Pharmacy, University of Porto, Rua de Jorge Viterbo Ferreira, 228, 4050-313 Porto, Portugal; 3 FP-I3ID, FP-ENAS/CEBIMED, Fernando Pessoa Energy, Environment, and Health Research Unit/Biomedical Research Center, Portugal and Faculty of Health Sciences, Fernando Pessoa University, 4200-150 Porto, Portugal; jcatita@ufp.edu.pt; 4 Paralab, AS, 4420-437 Gondomar, Portugal; hugo.goncalves@paralab.pt; 5 REQUIMTE/LAQV—Polytechnic of Porto, School of Engineering, Rua Dr. António Bernardino de Almeida, 4229-015 Porto, Portugal; ana.silva@graq.isep.ipp.pt (A.M.S.); francisca.rodrigues@graq.isep.ipp.pt (F.R.)

**Keywords:** blood–brain barrier (BBB), brain delivery, neurodegenerative diseases, amyotrophic lateral sclerosis (ALS), lipid nanoparticles, nanostructured lipid carriers (NLC), riluzole, lactoferrin, lactoferrin receptors, drug targeting

## Abstract

Amyotrophic lateral sclerosis (ALS) is a neurodegenerative disease with a very poor prognosis. Its treatment is hindered by a lack of new therapeutic alternatives and the existence of the blood–brain barrier (BBB), which restricts the access of drugs commonly used in ALS, such as riluzole, to the brain. To overcome these limitations and increase brain targeting, riluzole-loaded nanostructured lipid carriers (NLC) were prepared and functionalized with lactoferrin (Lf), facilitating transport across the BBB by interacting with Lf receptors expressed in the brain endothelium. NLC were characterized with respect to their physicochemical properties (size, zeta potential, polydispersity index) as well as their stability, encapsulation efficiency, morphology, in vitro release profile, and biocompatibility. Moreover, crystallinity and melting behavior were assessed by DSC and PXRD. Nanoparticles exhibited initial mean diameters between 180 and 220 nm and a polydispersity index below 0.3, indicating a narrow size distribution. NLC remained stable over at least 3 months. Riluzole encapsulation efficiency was very high, around 94–98%. FTIR and protein quantification studies confirmed the conjugation of Lf on the surface of the nanocarriers, with TEM images showing that the functionalized NLC presented a smooth surface and uniform spherical shape. An MTT assay revealed that the nanocarriers developed in this study did not cause a substantial reduction in the viability of NSC-34 and hCMEC/D3 cells at a riluzole concentration up to 10 μM, being therefore biocompatible. The results suggest that Lf-functionalized NLC are a suitable and promising delivery system to target riluzole to the brain.

## 1. Introduction

Amyotrophic lateral sclerosis (ALS) is an invariably fatal neurodegenerative disease. It is characterized by the degeneration of both upper and lower motor neurons in the brain and spinal cord [1]. Degeneration and gliosis of the axons leads to denervation and progressive muscle atrophy, with a median survival of 2 to 5 years after onset [1,2]. The etiology of ALS is not well-defined. It is a heterogeneous disease caused by complex biological, genetic, and environmental factors. Several molecular and cellular mechanisms have been implicated, including genetic mutations (e.g., superoxide dismutase type 1 (SOD1) mutations), mitochondrial dysfunction, intracellular protein aggregates, free radical oxidative stress, excitotoxicity, inflammation, etc. [1,3].

Currently, only two drugs, riluzole and edaravone, are approved for ALS, providing modest benefits in function and/or mortality [3]. Riluzole is commercialized in the form of tablets, as an oral suspension, or as an oral film, whereas edavarone is marketed as a solution for intravenous infusion [4]. Despite various ongoing Phase III ALS pharmacological trials (for instance, masitinib, arioclomol, levosimendan, or tofersen), the existence of physical barriers (e.g., the blood–brain barrier (BBB)), clinical heterogeneity, and lack of clear biological targets pose significant challenges in establishing new treatments [3,5]. To address the considerable socioeconomic burden of this disease, the development of novel therapeutic strategies is urgently needed [5,6]. In this regard, nanotechnology-based approaches may be useful to increase bioavailability and improve ALS drug targeting to the brain. Nanocarriers can efficiently protect and transport therapeutic agents across the BBB, avoiding extensive systemic distribution [5,7]. Currently, there are five clinical trials in progress in which nanocarriers are being tested for ALS. They all concern the same formulation—gold nanocrystals in an oral bicarbonate solution (denominated CNM-Au8) [8,9,10,11,12].

Lipid-based nanocarriers such as liposomes, solid lipid nanoparticles (SLN), nanostructured lipid carriers (NLC), and nanoemulsions are considered promising brain delivery systems over other nanocarriers. The use of biodegradable lipids and GRAS (generally recognized as safe) excipients confers them high biocompatibility and low cytotoxicity, which are advantageous for brain delivery. Their lipophilic nature facilitates crossing into the central nervous system (CNS) [7]. Furthermore, their surfaces can be modified with several coating materials (e.g., polysorbate 80) or ligands (e.g., transferrin, lactoferrin, peptides, etc.), which, by adsorbing serum plasma-penetrating proteins or by interacting directly with receptors and transporters of the BBB, can promote and increase the selectivity of the uptake [5,7]. Despite the well-documented potential of lipid nanoparticles in combating neurodegeneration, existing literature on the use of these nanocarriers for ALS treatment is very limited. Only a handful of studies have been reported [13,14,15,16,17].

The aim of this study was to develop lactoferrin (Lf) functionalized NLC to enhance riluzole delivery to the brain and improve targeting efficiency. Lf is a glycoprotein that belongs to the transferrin family. Lf receptors are overexpressed on brain endothelial cells of the BBB, intervening in receptor-mediated transcytosis [18,19]. Some authors have already demonstrated the superiority of nanocarriers functionalized with Lf when compared to nonfunctionalized ones, improving drug uptake through the BBB [20,21].

NLC were characterized regarding their physicochemical characteristics and stability. Functionalization was confirmed by Fourier transform infrared spectroscopy (FTIR) and protein quantification methods. To assess the potential efficacy of NLC for ALS treatment, in vitro cell viability assays on hCMEC/D3 cell line and on the motor neuron-like NSC-34 cell line were performed. To the best of our knowledge, this is the first study that reports the use of functionalized NLC for ALS.

## 2. Materials and Methods

### 2.1. Materials

Precirol^®^ATO5 (glyceryl distearate/glyceryl palmitostearate) was kindly provided by Gattefossé (Nanterre, France), and Kolliphor^®^ P188 micro Geismar (Poloxamer 188), by BASF (Ludwigshafen am Rhein, Germany). Mygliol^®^ 812 (triglycerides of capric/caprylic acids), Tween^®^ 80 (polysorbate 80), and stearic acid were acquired from Acofarma (Madrid, Spain), and riluzole (purity > 98%), from Beantown Chemical (Hudson, NH, USA). N-hydroxysuccinimide (NHS), N-(3-Dimethylaminopropyl)-N’-ethylcarbodiimide hydrochloride (EDC), and recombinant human lactoferrin were purchased from Sigma-Aldrich (Steinheim, Germany).

Dulbecco’s Modified Eagle Medium (DMEM), fetal bovine serum (FBS), Hank’s balanced salt solution (HBSS), nonessential amino acids, penicillin, streptomycin, and trypsin–EDTA were obtained from Invitrogen Corporation (Life Technologies, S.A., Madrid, Spain). EndoGRO^TM^ basal medium and EndroGRO-MV Complete Culture Media Kit were obtained from Merck (Darmstadt, Germany). Dimethyl sulfoxide (DMSO) and Triton X-100 were purchased from Sigma-Aldrich (Steinheim, Germany). All the other reagents were of analytical grade and used as supplied. The water used in all experiments was purified as obtained from a Milli-Q^®^ Direct 3 UV-R system (Millipore, Darmstadt, Germany).

### 2.2. Preparation of the Lipid Nanoparticles

NLC were produced by a hot homogenization technique followed by ultrasonication [22,23]. Precirol^®^ATO5 and Mygliol^®^ 812 were used as solid and liquid lipids, respectively. Both lipids have GRAS status [24]. A blend of mono-, di- and triglycerides, Precirol^®^ ATO5 leads to the formation of a lattice with an imperfect arrangement, thus providing more space to incorporate the drug and avoid its expulsion [24]. Polysorbate 80 and poloxamer 188 were chosen as nonionic surfactants. Compared with ionic surfactants, stabilizers of this type have a very low toxic and irritation potential [25]. Moreover, they produce particles with smaller sizes, also having the advantage of enhancing brain uptake [24]. Indeed, polysorbate 80 and poloxamer 188 have been repeatedly demonstrated to be able to increase drug transport to the brain through different administration routes, by coating on the surface of the nanocarriers [26]. To facilitate the posterior functionalization of the NLC with Lf, stearic acid was also incorporated as excipient.

Briefly, the lipid and aqueous phases were first heated separately at about 80 °C, with the aqueous phase being added to the molten lipid mixture. The preemulsion was then homogenized with an Ultra-Turrax T25 with S 25 N–18 G dispersing element (Janke and Kunkel IKA-Labortechnik, Staufen, Germany) at 8000 rpm for 5 min; this was followed by sonication for 15 min at an amplitude of 70% with a probe sonicator (Vibro Cell VCX 130, 6 mm probe, Sonics & Materials, Newtown, CT, USA). The obtained oil-in-water nanoemulsion was transferred to glass vials and cooled down in an ice bath for about 20 min, with the NLC being formed by lipid recrystallization. The composition of the different NLC is described in Table 1.

### 2.3. Functionalization of NLC with Lactoferrin

Functionalization of NLC with Lf was achieved by carbodiimide chemistry, following a previously reported method with some minor modifications [20]. Lf was covalently coupled by its amino group to the carboxylic acid group of the stearic acid present on the surface of riluzole-loaded NLC (NLC Riluzole), with EDC and NHS serving as activators of the carboxylic group [27].

Briefly, 10 mL of riluzole-loaded NLC was incubated with 22.5 mL of a Lf solution (1 mg/mL of Lf in phosphate-buffered saline (PBS), pH 7.4) at room temperature for 4 h with gentle stirring, in the presence of EDC and NHS. To remove excessive unbound Lf and any other by-products, the functionalized NLC were then centrifuged (Allegra^®^ X-15R Centrifuge, Beckman Coulter, Brea, CA, USA) at 5000× *g* for 1 h using Amicon^®^ Centrifugal Filter Units with a cutoff of 100 kDa.

### 2.4. Physicochemical Characterization and Stability of NLC

To characterize and study the stability of NLC, the particle size, polydispersity index (PDI), and zeta potential (ZP) were monitored at different time points (day 0, 1 month, 2 months, and 3 months) after production and storage in closed glass vials at both 5 ± 1 °C and 25 ± 1 °C. Furthermore, encapsulation efficiency (EE%) was also assessed.

#### 2.4.1. Particle Size, PDI, and ZP Analysis

The mean hydrodynamic diameter and PDI were determined by dynamic light scattering (DLS) using a particle size analyzer (Brookhaven Instruments, Holtsville, NY, USA). Zeta potential was estimated using the same equipment by electrophoretic light scattering (ELS). All samples were previously diluted in milli-Q water (dilution 1:100), and measurements were performed with a light incidence angle of 90°.

#### 2.4.2. Encapsulation Efficiency (EE)

The EE (%) of NLC Riluzole and functionalized NLC were assessed by high-performance liquid chromatography (HPLC) (Dionex UltiMate™ 3000, Thermo Scientific, Waltham, MA, USA), following a method that was developed in house. The chromatographic analysis was performed at 263 nm, using a BDS Hypersil^TM^ C18 column (150 mm × 4.60 mm internal diameter, 5 µm particle size; Thermo Scientific, Waltham, MA, USA), and methanol/water pH 3, 70:30 (*v*/*v*), as the mobile phase, eluted at a flow rate of 1 mL/min. Injections were made in triplicate (*n* = 3) with a sample volume of 10 µL. A calibration curve was built up by analyzing independent standard solutions and fitting the respective data to the least squares linear regression, which gave a correlation coefficient (R) of 0.9994.

Briefly, 2 mL of freshly prepared formulation was diluted in 10 mL of Milli-Q^®^ water and filtered through a 5 µm cellulose nitrate membrane (Sartorius, Gottingen, Germany) to remove unencapsulated drug crystals. Afterwards, 2 mL of the diluted formulation was added to 8 mL of ethanol and thoroughly mixed in order to extract the riluzole from the lipid matrix. The mixture was centrifuged for 15 min at 5000 rpm (Thermo Scientific^TM^ Heraeus^TM^ Multifuge X1R Refrigerated Benchtop Centrifuge, Waltham, MA, USA), and the supernatant was collected, filtrated using a 0.45 µm PTFE syringe filter (Millipore, Germany) [28], and analyzed by HPLC. The EE of the NLC was determined by calculating the amount of riluzole in the supernatant of the filtered formulations as follows:

(1) $$\mathrm{EE}\;\left(\%\right)=\frac{\mathrm{Amount}\;\mathrm{of}\;\mathrm{riluzole}\;\mathrm{in}\;\mathrm{the}\;\mathrm{filtered}\;\mathrm{formulation}}{\mathrm{Total}\;\mathrm{amount}\;\mathrm{of}\;\mathrm{riluzole}}\times 100$$

### 2.5. Transmission Electron Microscopy (TEM)

The shape and morphology of the functionalized NLC were examined by transmission electron microscopy (TEM). First, 10 µL of the NLC dispersion was mounted on Formvar/carbon film-coated mesh nickel grids (Electron Microscopy Sciences, Hatfield, PA, USA) and left standing for 2 min. The liquid in excess was removed with filter paper, and afterwards, 10 µL of 1% uranyl acetate was added on to the grids for 10 s for negative staining. Visualization was carried out on a JEOL JEM 1400 TEM at 80 kV (Tokyo, Japan). Images were digitally recorded using a CCD digital camera Orious 1100W (Tokyo, Japan).

### 2.6. In Vitro Drug Release Studies

The in vitro release profiles of NLC Riluzole and functionalized NLC were determined using the dialysis bag technique [29]. The dialysis bag (molecular weight cutoff: 3.5 kDa; Cellu•Sep^®^, Montluçon, France) was soaked in distilled water for 12 h prior to use.

Briefly, 5 mL of either NLC Riluzole or functionalized NLC was put in the dialysis bag, clamped, and incubated in 200 mL of PBS pH 7.4 in a water bath at 37.0 ± 0.5 °C, under continuous magnetic stirring (450 rpm). At specified time intervals (0.25, 0.50, 1, 2, 4, 6, 8, 10, 24, 27, 29, 30, 34, 48, and 60 h), 2 mL of aliquot was withdrawn, with no medium replacement. The samples were filtered through a 0.45 µm membrane filter, and the amount of riluzole released was quantified by HPLC as stated before. Measurements were carried out in triplicate (*n* = 3), and the results were expressed as % cumulative drug release at each time point (mean ± SD).

To characterize the transport mechanisms at play, the release profiles were fitted to various kinetic models, namely, zero-order (Equation (2)), Higuchi (Equation (3)), and Korsmeyer–Peppas (Equation (4)) [30]:

(2) $$Q_{t}={\;Q}_{0}+{\;K}_{0}t\;\;\;$$

(3) $$Q_{t}={\;Q}_{0}+{\;K}_{H}t^{0.5}$$

(4) $$Q_{t}={\;Q}_{0}+{\;K}_{\mathrm{KP}}t^{n}$$

where Q_t_ is the amount of riluzole dissolved in time t, Q_0_ is the amount of riluzole in the dissolution medium in the time 0, K_0_ is the zero-order release constant, K_H_ is the Higuchi dissolution constant, K_KP_ is the Korsmeyer–Peppas release constant, and n is the latter’s release exponent.

For model fitting, only the first 60% of the drug release was considered [31]. The best fit for the release data is usually evaluated by the coefficient of determination (R^2^) [32]. However, R^2^ tends to increase with the addition of more parameters. Therefore, when comparing kinetic models that have different numbers of parameters, the adjusted coefficient of determination ($R_{\mathrm{adj}}^{2}$) is more appropriate [32]. $R_{\mathrm{adj}}^{2}$ can be calculated from Equation (5):

(5) $$R_{\mathrm{adj}}^{2}=1-\frac{\left(1-R^{2}\right)\left(q-1\right)}{q-p-1}$$

where q is the number of experimental data points and p is the number of parameters.

The model with the highest $R_{\mathrm{adj}}^{2}$ was considered as the best fitting model [32].

### 2.7. Lactoferrin Conjugation Efficiency

The Bradford assay was employed to quantify the Lf conjugated on the surface of the NLC [20]. After production, the nanoparticles were centrifuged (Allegra^®^ X-15R Centrifuge, Beckman Coulter, Brea, CA, USA) at 5000× *g* for 1 h, using Amicon^®^ Centrifugal Filter Units (cutoff of 100 kDa). Following incubation with the Bradford reagent, the amount of unbound Lf within the supernatant was measured by reading the absorbance at 595 nm using a UV–Vis spectrophotometer (Jasco V-650 spectrophotometer, Easton, MD, USA) [20]. The experiments were performed in triplicate (*n* = 3), and conjugation efficiency was expressed as percentage of Lf bound to the NLC.

### 2.8. Fourier Transform Infrared (FTIR) Spectroscopy

To confirm the functionalization of the NLC with Lf and infer about possible drug–lipid interactions, Fourier transform infrared spectroscopy (FTIR) was performed. The functionalized NLC were previously frozen overnight at −85 °C and lyophilized at −75 °C and 0.4 mBar using a LyoQuest freeze dryer (Telstar, Terrassa, Spain).

The infrared spectra were obtained by placing the samples (riluzole, Precirol^®^ ATO5, molten mixture of the lipids and drug—drug–lipid melt, and functionalized NLC) on a PerkinElmer Frontier™ FTIR Spectrometer (Waltham, MA, USA) equipped with a universal attenuated total reflectance (ATR) attachment and a diamond crystal. For each measurement, 32 scans at a resolution of 4 cm^−1^ were accumulated at frequencies between 4000 to 600 cm^−1^.

### 2.9. Differential Scanning Calorimetry (DSC)

To study crystallinity and melting behavior, DSC analysis was performed using a DSC 214 Polyma^®^ (NETZCH, Selb, Germany). Mygliol^®^ 812, Precirol^®^ ATO5, riluzole, drug–lipid melt, and freeze-dried functionalized NLC (10–20 mg) were put into aluminum crucibles that were perforated on top. An empty aluminum crucible was used as reference.

The thermal program included two cycles of heating/cooling. The first cycle, recorded with a scan rate of 10 °C/min, involved one cooling to 10 °C followed by an isotherm for 5 min, a heating from 10 to 160 °C, and another cooling to 10 °C. Between cycles, an isotherm at 10 °C for 5 min was performed. The second cycle comprised a heating from 10 to 160 °C (at a rate of 10 °C/min) followed by cooling to 25 °C (at a rate of 20 °C/min). Data were obtained using the Proteus^®^ 8.0.1 software (NETZCH, Selb, Germany).

### 2.10. Powder X-ray Diffraction (PXRD)

X-ray diffractograms for Precirol^®^ ATO5, riluzole, drug–lipid melt, and freeze-dried functionalized NLC were acquired using a Miniflex 600 X-ray diffractometer (Rigaku, Tokyo, Japan) equipped with Cu-Kα radiation. The instrument was set at a current of 15 mA and a voltage of 40 kV. The samples were scanned from 3 to 60° (2θ) with a scanning rate of 5 °/min and a step size of 0.01°.

### 2.11. Cell Culture

Mouse motor neuron-like hybrid cells (NSC-34 cell line) were obtained from Cedarlane (Hornby, ON, Canada). The cells were grown in DMEM supplemented with inactivated FBS (10%, *v*/*v*), L-glutamine (1%, *v*/*v*), nonessential amino acids (1%, *v*/*v*) and antibiotic–antimycotic mixture (1%, *v*/*v*; final concentration of 100 U/mL penicillin and 100 U/mL streptomycin).

Immortalized human cerebral microvascular endothelial cells (hCMEC/D3 cell line) were purchased from Cedarlane (Hornby, ON, Canada). The cells were grown in EndoGRO^TM^ basal medium supplemented with FBS (5%, *v*/*v*), rhEGF (5 ng/mL), L-glutamine (10 mM), ascorbic acid (50 μg/mL), EndoGRO-LS supplement (0.2%, *v*/*v*), hydrocortisone hemisuccinate (1.0 μg/mL), and heparin sulfate (0.75 U/mL).

Cells were maintained in an incubator (Cell Culture^®^ CO_2_ incubator, ESCO GB Ltd., Barnsley, England, UK) at 37 °C in a water-saturated atmosphere with 5% CO_2_ and subcultured every 2–3 days using trypsin–EDTA to detach them from the flasks. The culture medium was replaced every other day.

### 2.12. MTT Cytotoxicity Assay

The vital mitochondrial dye 3-(4,5-dimethylthiazol-2-yl)-2,5-diphenyltetrazolium bromide (MTT) assay was performed, following the methodology described by Pinto et al. [33], in order to evaluate the cytotoxic effect of the different formulations on the two cell lines. Passages 44–45 and passages 4–6 were used for the hCMED/D3 and NSC-34 cell lines, respectively.

Briefly, cells were seeded in 96-well plates (25 × 10^3^ cells/mL) and exposed to different concentrations (0.1, 1, and 10 µg/mL) of NLC Placebo, NLC Riluzole, functionalized NLC, and free riluzole for 24 h. Following the removal of the formulations from each well, cells were washed with HBSS. The number of viable cells was determined by adding MTT reagent and incubating for 3 h at 37 °C. DMSO was used to solubilize the crystals. Triton X-100 1% (*w*/*v*) and culture medium were used as negative and positive controls, respectively. The absorbance was read at 590 nm with background subtraction at 630 nm. Results were expressed as percentages of cell viability.

## 3. Results and Discussion

### 3.1. Physicochemical Characterization and Stability Studies

Assessing the physicochemical properties of the nanoparticles is essential to determine their biological fate, as well as their passage mechanism across the BBB [34].

The characterization of NLC is summarized in Table 2, where the mean diameter, PDI, zeta potential, and EE are presented.

**Figure 1:**
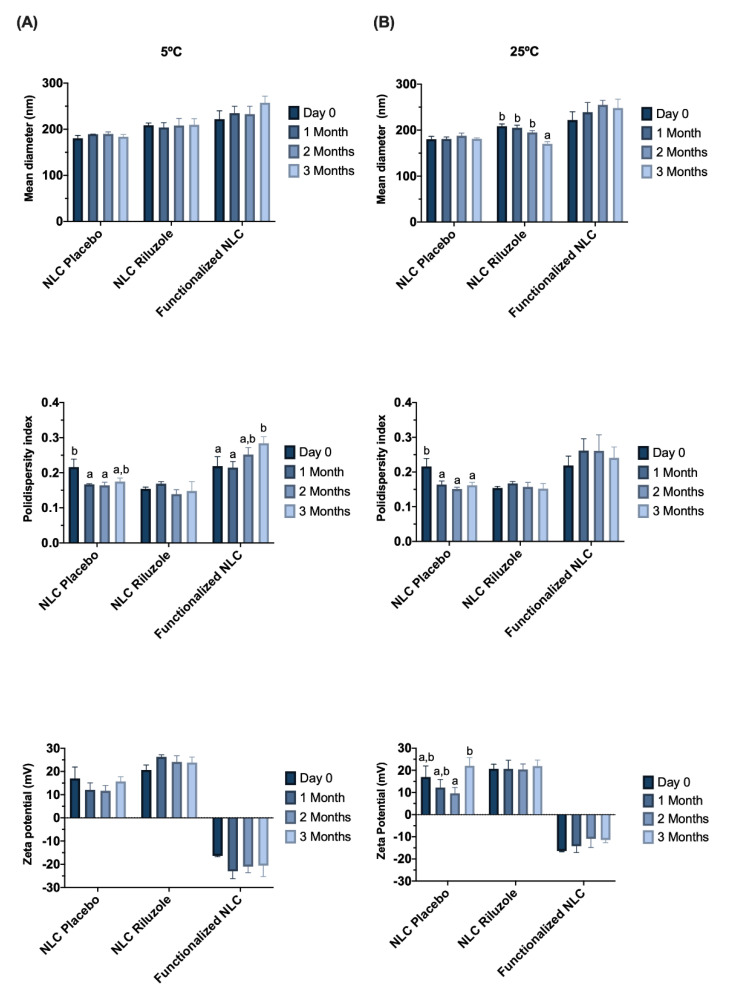
Effect of storage time on particle size, PDI, and zeta potential for NLC Placebo, NLC Riluzole, and functionalized NLC over 3 months at 5 °C (**A**) and 25 °C (**B**). Values are expressed as the mean ± SD (*n* = 3). Data were analyzed using Tukey’s HSD test, with different letters (a,b) representing statistically significant differences (*p* < 0.05) for the same formulation at different time points.

Stability studies were also performed by monitoring variations in the size, PDI, and ZP of the NLC, which were stored at 5 °C and 25 °C, for 3 months (Figure 1).

Although, for functionalized nanoparticles, the surface coating seems to be the most crucial determinant for BBB crossing [35], size should always be taken into consideration, given that smaller particles tend to penetrate better into the brain [34]. NLC showed an initial mean diameter lower than 220 nm (Table 2), with the particle size remaining below 250 nm for all formulations (Figure 1), which is an important characteristic that enables permeation through the BBB [23]. Several studies have demonstrated that different nanocarriers in this size range can effectively traverse the BBB. For instance, Pinheiro et al. developed quercetin-loaded SLN and NLC functionalized with transferrin [23] or RVG29 [22] (all particles with sizes below 250 nm); Zensi et al. [36] formulated albumin nanoparticles attached with apolipoprotein A-I, with an average size of 250–270 nm; Tosi et al. [37] produced poly(lactic-co-glycolic acid) (PLGA) polymeric nanoparticles modified with a mutated form of diphtheria toxin (CRM197), with sizes of around 220 nm; and Gu et al. [38] developed antibody-modified chitosan nanoparticles for the delivery of siRNA with a mean size of 235.7 nm.

The incorporation of riluzole and functionalization with Lf did not directly influence the size of the lipid nanoparticles, since no statistically significant differences were found between NLC Placebo and NLC Riluzole, nor between NLC Riluzole and functionalized NLC (Table 2). Furthermore, all the formulations remained stable throughout the storage period at each analyzed temperature (Figure 1), given that the average particle size had no tendency to increase.

Polydispersity index is a measure of the uniformity of particle size distribution. The formulations showed PDI values below 0.3 (Table 2), which for lipid nanoparticles suggests a fairly narrow and homogeneous size distribution with low variability [23,39]. Notably, there was an increase in the PDI of the functionalized NLC over time at 5 °C (*p* = 0.026) (Figure 1). Nevertheless, the value remained below 0.3, implying that any aggregation phenomena that may occur are minimal.

The zeta potential refers to the overall surface charge of a particle and is an indicator to predict stability over time. It is known that nanoparticles exhibit electrostatic stability and low propensity to aggregate when ZP is equal or greater than |30| mV [40]. NLC Placebo and NLC Riluzole presented positive ZP values between 16 and 20 mV. The presence of the drug did not have any impact in this parameter (*p* = 0.505) (Table 2). However, the addition of Lf led to an inversion of the ZP, with the NLC acquiring a negative charge of about −16 mV. This inversion in the charge is an indicator that the functionalization occurred. Despite the initial values not being as high as desired, the ZP of the formulations did not change significantly over time at either temperature (Figure 1).

The encapsulation efficiency was very high (94–98%) for all formulations, suggesting that functionalization did not affect the ability to efficiently incorporate the drug within the lipid nanoparticles.

Finally, the effect of the different temperatures (5 °C vs. 25 °C) on the various physicochemical properties was assessed at the end of the storage period (3 months). The only statistically significant difference regarded the size of the NLC Riluzole (*p* = 0.017).

Overall, the results of the stability studies appear to suggest that the NLC remain stable for at least 3 months.

### 3.2. In Vitro Release Studies

**Figure 2:**
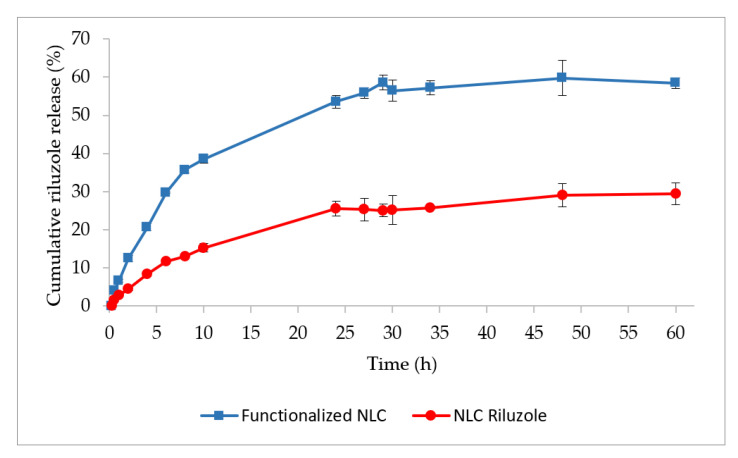
In vitro dissolution profiles of functionalized NLC and NLC Riluzole in PBS pH 7.4.

The release profiles of NLC Riluzole and functionalized NLC are shown in Figure 2. 

Both formulations exhibited a gradual and sustained release of riluzole for 10 h, followed by a plateau phase. At the end of the assay (t = 60 h), only about 30% of the drug was released from the NLC Riluzole. This could be explained by the nature of the drug. Riluzole is a BCS (Biopharmaceutical Classification System) class II drug, being a lipophilic compound with very low aqueous solubility and a high affinity for the lipids that constitute the NLC [41,42].

In contrast, the functionalized NLC released twice as much, with a cumulative release around 58% after 60 h. Sebastiani et al. [43] demonstrated that the binding of proteins on the surface of lipid nanoparticles can induce a redistribution of the lipids at the shell and the core, which also impacts the internal structure of the nanoparticles, causing the release of the encapsulated compound. Therefore, the incorporation of Lf could have resulted in a structural rearrangement of the lipids within the lipid matrix, leading to a higher and faster drug diffusion to the PBS medium. Moreover, Lf has amphiphilic domains, possessing both hydrophilic and hydrophobic regions [44,45,46]. It is possible that the hydrophilic regions of Lf exposed on the functionalized NLC also facilitated the interaction with the solvent molecules and released the riluzole molecules entrapped closer to the surface [47].

The results for the kinetic modeling of the riluzole release profiles from the NLC are presented in Table 3.

Both formulations showed good correlation to the Higuchi and Korsmeyer–Peppas equations ($R_{\mathrm{adj}}^{2}>0.99)$. Ultimately, the Higuchi model provided the best fit for the NLC (highest $R_{\mathrm{adj}}^{2}$).

As shown in Table 3, the drug release exponent (n) was 0.5548 and 0.5579 for NLC Riluzole and functionalized NLC, respectively. Given that 0.45 < n < 0.89, the release can be categorized as anomalous or non-Fickian diffusion, where a combined pattern of both diffusion and lipid erosion may be involved in the transport mechanism [48,49]. Nonetheless, since the value of n is closer to the lower range of the interval (i.e., to 0.45), it can be considered that the release is mainly governed by diffusion.

### 3.3. Morphology Determination

**Figure 3:**
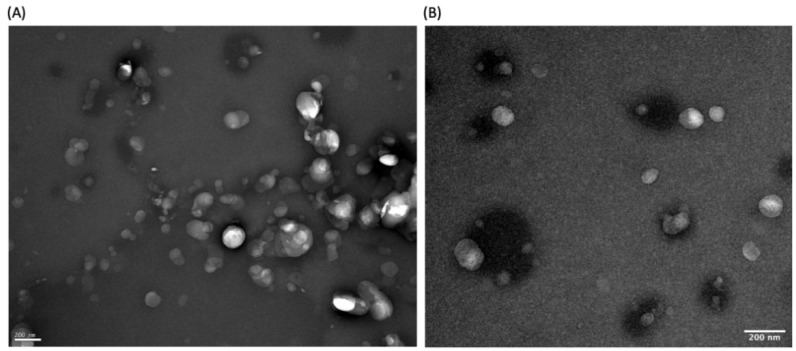
(**A**,**B**) High-resolution TEM images of different sections of freshly prepared functionalized NLC. Magnification of 50,000×.

TEM images (Figure 3A,B) showed that the functionalized NLC have a size around 200 nm, seemingly confirming the results obtained by DLS. Furthermore, the micrographs also revealed that the NLC were spheric and uniform in shape, with a smooth and round surface, and that there was no visible aggregation of nanoparticles, which is a good indicator of the stability of the formulations.

### 3.4. Lactoferrin Conjugation Efficiency

The conjugation efficiency was assessed by the Bradford assay. Previous works with Lf-functionalized nanocarriers have generally had conjugation efficiencies around 70% or lower. For instance, Singh et al. [20] reported Lf-SLN with a conjugation efficiency of 71%, and Huang et al. [50] produced Lf-liposomes with a protein conjugation efficiency of 74%. Both systems were intended for brain delivery.

The functionalized NLC obtained in the present study surpassed these values, with a coupling efficiency estimated at 95.3 ± 0.3%. This indicates that the conjugation process was very efficient and that the developed NLC could, in theory, lead to a more efficient targeting and brain uptake, since more Lf is available to interact with BBB endothelial cell receptors.

### 3.5. FTIR Spectroscopy

**Figure 4:**
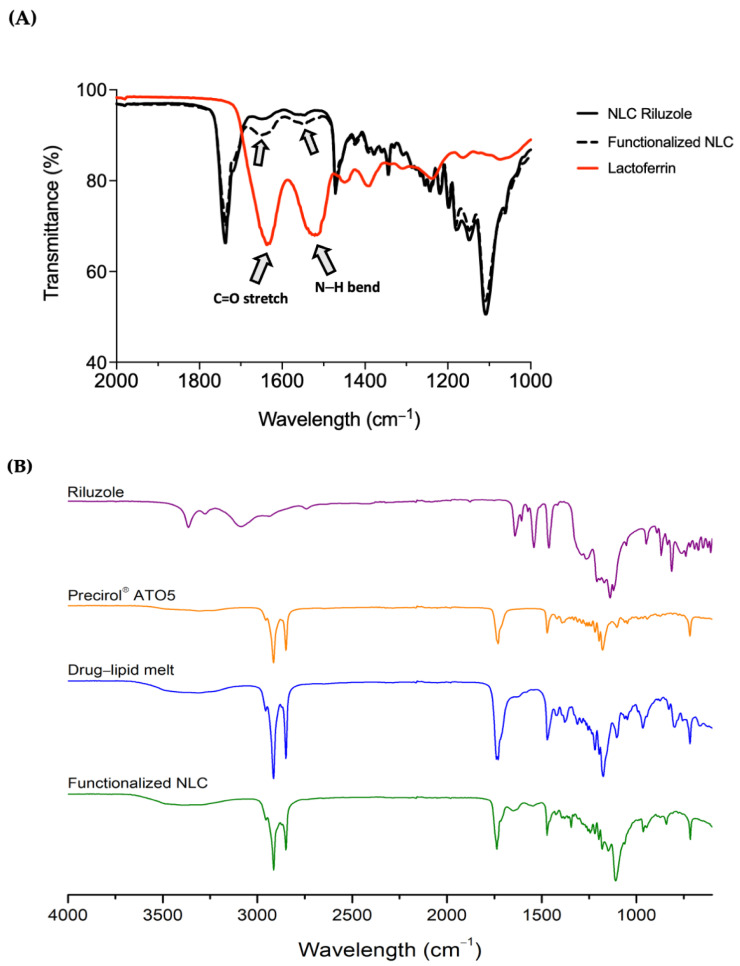
(**A**) Infrared spectra for NLC before and after functionalization with Lf protein. Note: Lf was used as a reference to compare with the functionalized nanoparticles. To show the bands of interest, the wavelength scale was limited to the range of 1000 to 2000 cm^−1^; (**B**) full infrared spectra of riluzole, Precirol^®^ ATO5, drug–lipid melt, and functionalized NLC.

The functionalization of NLC was assessed by FTIR analysis. Figure 4A depicts two main bands in the spectrum of the Lf sample, which are also present in the functionalized nanoparticles, specifically, the bands at around 1630 and 1510 cm^−1^, corresponding to the C=O stretching and N–H bending vibrations of the amide I and amide II functional groups of the Lf protein, respectively [21,51]. The amide I bond of Lf was recorded at 1628 cm^−1^ and underwent a shift to 1640 cm^−1^ in the functionalized NLC, whereas the amide II bond shifted from 1510 to 1538 cm^−1^. These findings seem to confirm that Lf was conjugated on the surface of the nanoparticles through covalent interactions. Such peaks appear as well in the spectrum of NLC Riluzole, although in this case, they can be attributed to the presence of the liquid lipid Miglyol^®^ 812, which has a chemical structure containing N–H and C=O bonds [23]. Moreover, in comparison with those of the functionalized NLC, the intensity of the peaks was lower, which further corroborates that the conjugation of the nanoparticles with Lf was indeed successful [23].

To gain a better understanding on the state of the drug in the NLC, in Figure 4B is displayed the full FTIR spectrum of bulk riluzole, Precirol^®^ ATO5, drug–lipid melt, and the functionalized NLC. Precirol^®^ ATO5 presented several characteristic peaks, identified at 1470 cm^−1^ (C–C stretching), 1730 cm^−1^ (C=O stretching), and 2850 and 2914 cm^−1^ (C–H stretching), as reported elsewhere [52,53]. Riluzole showed two bands at 3266 and 3360 cm^−1^ (N–H stretching), confirming the presence of the primary amine group [41]. Additionally, the peaks at 814 and 868 cm^−1^ (C–H bending), as well those at 1460 cm^−1^ (C=C stretching), 1550 cm^−1^ (C–H in plane bending) and 1640 cm^−1^ (C=N stretching), can all be attributed to different vibrations of its benzothiazole aromatic ring [54,55]. The molecular signatures of the drug–lipid melt and the functionalized NLC were comparable to that of Precirol^®^ ATO5. This was to be expected, given the high lipid content compared to the drug. This also demonstrates the successful entrapment of the drug in the lipid nanoparticles, since the majority of absorption bands ascribed to riluzole were not visible in the NLC spectrum, and those that were, were of lower intensity.

### 3.6. DSC

**Figure 5:**
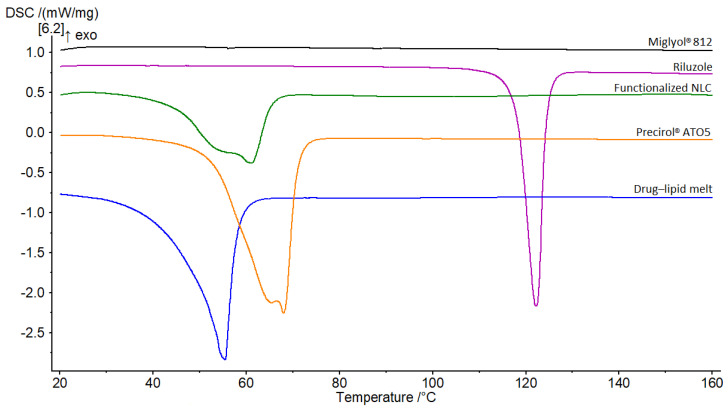
DSC thermograms of Miglyol^®^ 812, riluzole, functionalized NLC, Precirol^®^ ATO5 and, drug–lipid melt.

The overlay of the thermograms for the first DSC cycle is presented in Figure 5. Riluzole demonstrated a single sharp peak indicative of a crystalline compound. It occurred at 122.1 °C, which corresponds to the melting point of riluzole [41]. Bulk solid lipid Precirol^®^ ATO5 displayed a peak at 68 °C, with a small shoulder of lower enthalpy at around 65 °C. These two distinct melting events are attributable to the presence of different polymorphic forms that occur in complex mixtures of glycerides such as Precirol^®^ ATO5 [56]. This phenomenon has been well reported in literature, with the melting event of lower temperature corresponding to the metastable α-polymorph of the lipid and the major peak to its stable β-form [57]. Concerning Miglyol^®^ 812, its thermogram did not show any thermal event, since this excipient is an oil at room temperature [58].

The riluzole peak was not observed in the thermogram of the melt mixture, nor in that of the functionalized NLC. This suggests that (1) the drug was well solubilized in the molten lipids and (2) it was encapsulated in an amorphous or molecularly dispersed state within the nanoparticles [56,59]. For both thermograms, the endothermic peaks were broader, accompanied by a shift of the onset and the melting point to lower temperatures. The shift was slightly more pronounced in the case of the drug–lipid melt, with the melting point demonstrating a 12 °C drop compared to that of the solid lipid (from 68 °C to 56 °C). Moreover, there was a sharp decline in the melting enthalpies (ΔH). These differences are mainly due to the interactions of the solid lipid with the liquid lipid and the surfactants during the preparation process [57], which lead to a less ordered lattice that is favorable for encapsulating greater amounts of drug [53,59]. However, a certain effect due to the nanometric size of the NLC should be taken into account as well [57]. Bunjes et al. demonstrated that particles with smaller sizes lead to a high surface area, creating an energetically suboptimal state that causes depression of the melting point [60].

### 3.7. PXRD Analysis

**Figure 6:**
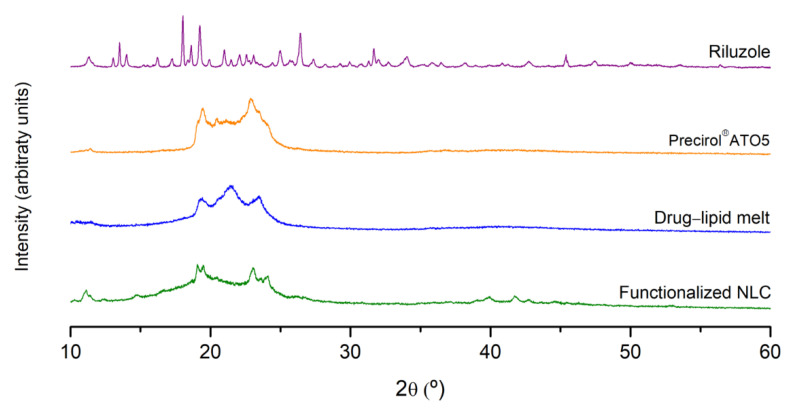
X-ray diffractograms of riluzole, Precirol^®^ ATO5, drug–lipid melt, and functionalized NLC.

To further investigate possible changes in the crystalline structure of the nanoparticles, PXRD experiments were carried out. The diffraction patterns of riluzole, Precirol^®^ ATO5, drug–lipid melt, and functionalized NLC are displayed in Figure 6.

Riluzole exhibited several characteristic sharp peaks at different diffraction angles (2θ of 13.5°, 18.0°, 19.3°, 21°, 25°, 26.4°, 31.7°, 35°, and 45.4°), revealing the highly crystalline nature of the drug [55]. These peaks were absent in the diffractograms of the drug–lipid melt and of the functionalized NLC. In turn, the diffractograms essentially garnered aspects ascribed to the solid lipid, namely, the peaks at 19.5° and 23°. However, these peaks were wider and less intense compared to those of the lipid alone. Such findings indicate not only the successful encapsulation of the drug within the lipid matrix, but a reduction in the crystalline nature of riluzole and the lipid, forming a stabilized amorphous structure [61]. The results were in agreement with the FTIR and DSC data.

### 3.8. Cell Viability Assay

To validate the nanocarriers as a safe strategy for brain drug delivery, a MTT assay was undertaken to test the cell viability after exposure to the NLC formulations [22]. As the nanosystems were developed for brain targeting aiming at the treatment of ALS, it was important to assess their potential cytotoxicity not only in a BBB model, but also in the disease-affected cells, which in ALS are the motor neurons. Therefore, two cell lines were selected: NSC-34 and hCMEC/D3. NSC-34 is a hybrid cell line generated by the fusion of motor neurons from the spinal cords of mouse embryos with mouse neuroblastoma N18TG2 cells. These cells express many motor neuron-like properties and are the most used in ALS research [62,63]. On the other hand, hCMEC/D3 cell monolayer is one of the most reliable human BBB models, since it is easy to grow and reproducible and closely mimics the in vivo phenotype and permeability values [40,64].

**Figure 7:**
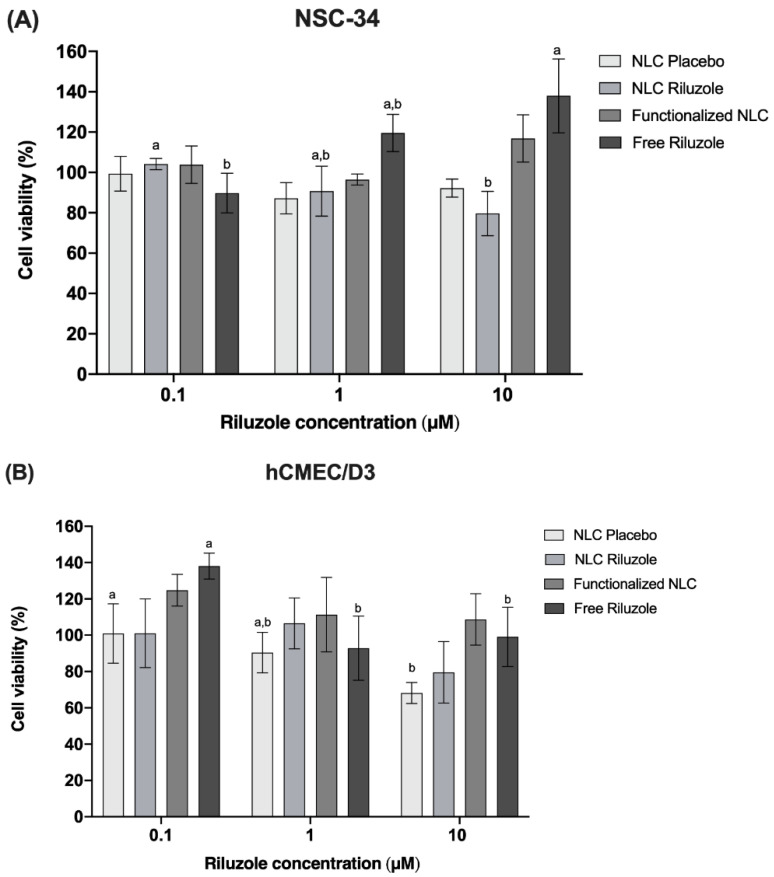
Effects of NLC Placebo, NLC Riluzole, functionalized NLC, and free riluzole exposure on the viability of NCS-34 (**A**) and hCMEC/D3 (**B**) cells at different concentrations (0.1–10 μM), as measured by the MTT assay. Values are expressed as mean ± SD (*n* = 6). Different letters (a,b) mean significant differences between concentrations of the same sample (*p* < 0.05) according to Tukey’s HSD test.

Figure 7 shows the viability, expressed as percentage of the positive control, induced in NSC-34 cells (A) and hCMEC/D3 cells (B) after 24 h of incubation in the presence of different concentrations (0.1, 1, and 10 μM) of NLC Placebo, NLC Riluzole, functionalized NLC, and free riluzole. Pure free riluzole was not toxic for the cell lines. It is possible to observe that the NLC did not produce any relevant cytotoxic effect on either cell line in the studied range, with viability remaining above 70% even at the highest concentration tested (10 μM). Therefore, the incorporation of riluzole in the nanocarriers and the functionalization of said nanocarriers did not seem to affect the cell integrity, indicating that the formulations have a good biocompatibility.

## 4. Conclusions

The advent of nanotechnology has thrust lipid nanoparticles such as NLC into the spotlight for the treatment of neurodegenerative diseases. Surface-modified nanocarriers can curtail the inherent constraints imposed by the BBB, enhancing drug delivery and uptake to the brain. Within this context, in this study, NLC were functionalized with a specific ligand, namely lactoferrin, to facilitate riluzole diffusion through the BBB and improve ALS treatment.

NLC exhibited physicochemical properties compatible with brain application (sizes below 250 nm, PDI below 0.3, and intermediate ZP values between |16| and |20| mV), having remained stable throughout at least 3 months of storage. DSC and PXRD confirmed the encapsulation of the drug inside the nanoparticles, which was corroborated by the high entrapment efficiency achieved (94–98%). FTIR and protein quantification studies seemed to demonstrate that the conjugation with Lf was successful. The MTT assay revealed no significant cytotoxicity of the NLC up to 10 μM of riluzole concentration. Overall, the results of the experiments suggest that the developed nanocarriers constitute a promising and safe brain targeting drug delivery system possessing good biocompatibility and stability.

Further research should be carried out to assess the permeability of the NLC in an in vitro BBB model and the associated transport mechanisms/internalization pathways, as well as to test their in vivo performance and efficacy in an ALS animal model.

## Figures and Tables

**Table 1:** Composition of the developed NLC (%, *w*/*w*).

Excipient	NLC Placebo	NLC Riluzole ^1^
Precirol^®^ ATO5	5.250	5.250
Miglyol^®^ 812	2.250	2.250
Stearic acid	0.125	0.125
Riluzole	—	0.100
Tween^®^ 80	2.000	2.000
Kolliphor^®^ P 188 micro	1.000	1.000
Ultra-purified water	89.000	88.900

^1^ The same composition was applied to produce the functionalized NLC.

**Table 2:** Mean diameter, polydispersity index, zeta potential, and encapsulation efficiency of NLC formulations on day 0. Values are expressed as the mean ± SD (*n* = 3).

	Mean Diameter (nm) ^1^	Polydispersity Index ^1^	Zeta Potential (mV) ^1^	Encapsulation Efficiency (%) ^2^
NLC Placebo	180.3 ± 6.3 ^a^	0.216 ± 0.023 ^b^	16.97 ± 4.98 ^b^	—
NLC Riluzole	208.5 ± 5.0 ^a,b^	0.154 ± 0.023 ^a^	20.67 ± 2.11 ^b^	98.70 ± 0.96 ^a^
Functionalized NLC	221.9 ± 18.1 ^b^	0.219 ± 0.027 ^b^	−16.41 ± 0.31 ^a^	94.21 ± 4.35 ^a^

^1^ Data were analyzed using Tukey’s HSD test, with different letters (^a,b^) representing statistically significant differences (*p* < 0.05) between the different formulations. ^2^ Data were analyzed using the *t*-Student’s test (statistical significance was considered for *p* < 0.05).

**Table 3:** Estimated kinetic parameters of the different mathematical models fitted to the riluzole release from NLC Riluzole and functionalized NLC.

Kinetic Model	Parameters	NLC Riluzole	Functionalized NLC
Zero-order	$K_{0}$	0.0254	1.2734
	R^2^	0.9681	0.9689
	$R_{\mathrm{adj}}^{2}$	0.9617	0.9626
Higuchi	$K_{H}$	0.7353	1.9151
	R^2^	0.9945	0.9951
	$R_{\mathrm{adj}}^{2}$	0.9933	0.9941
Korsmeyer–Peppas	$K_{\mathrm{KP}}$	0.4999	1.2734
	R^2^	0.9950	0.9957
	$R_{\mathrm{adj}}^{2}$	0.9925	0.9935
	n	0.5548	0.5579

## Data Availability

The data presented in this study are available on request from the corresponding author.
